# Molecular Detection of Tick-Borne Pathogens in American Bison (*Bison bison*) at El Uno Ecological Reserve, Janos, Chihuahua, Mexico

**DOI:** 10.3390/pathogens10111428

**Published:** 2021-11-04

**Authors:** Diana M. Beristain-Ruiz, Cuauhcihuatl Vital-García, Julio V. Figueroa-Millán, José J. Lira-Amaya, Javier A. Garza-Hernández, Juan R. Sánchez-Ayala, Samuel Flores-Ceballos, Carlos A. Rodríguez-Alarcón, Martha P. Olivas-Sánchez, Gabriel Pons-Monarrez

**Affiliations:** 1 Departamento de Ciencias Veterinarias, Universidad Autónoma de Ciudad Juárez, Anillo Envolvente y Estocolmo s/n Colonia Progresista AP 1729-D Cd. Juárez, Chihuahua CP 32310, Mexico; diana.beristain@uacj.mx (D.M.B.-R.); carrodri@uacj.mx (C.A.R.-A.); zoeveterinaria@gmail.com (G.P.-M.); 2 CENID-Salud Animal e Inocuidad, Instituto Nacional de Investigaciones Forestales, Agrícolas y Pecuarias, Cuernavaca-Cuautla 8534, Progreso, Jiutepec CP 62574, Mexico; figueroa.julio@inifap.gob.mx (J.V.F.-M.); lira.juan@inifap.gob.mx (J.J.L.-A.); 3 Departamento de Ciencias Químico-Biológicas, Universidad Autónoma de Ciudad Juárez, Anillo Envolvente y Estocolmo s/n Colonia Progresista AP 1729-D Cd. Juárez, Chihuahua CP 32310, Mexico; javier.garza@uacj.mx (J.A.G.-H.); polivas@uacj.mx (M.P.O.-S.); 4 Facultad de Medicina Veterinaria y Zootecnia, Universidad Nacional Autónoma de México, Ciudad Universitaria, México D. F. CP 04510, Mexico; vetrichys@hotmail.com (J.R.S.-A.); fcmasam@gmail.com (S.F.-C.)

**Keywords:** *Bison bison*, ticks, North American prairies, tick-borne diseases

## Abstract

American bison (*Bison bison*) is listed as near-threatened and in danger of extinction in Mexico. Recent studies have demonstrated the presence of several emerging pathogens at the Janos Biosphere Reserve (JBR), inhabited by one wild herd of American bison. Blood samples were collected from 26 American bison in the JBR. We tested for the presence of *Anaplasma marginale*, *Babesia bigemina*, *B. bovis*, *Borrelia burgdorferi sensu lato*, and *Rickettsia rickettsii* DNA using nested and semi-nested PCR protocols performing duplicates in two different laboratories. Results showed three animals (11.5%) positive for *B. burgdorferi s. l.*, three more (11.5%) for *Rickettsia rickettsii,* and four (19.2%) for *B. bovis*. Two individuals were co-infected with *B. burgdorferi s. l.* and *B. bovis*. We found no animals positive for *A. marginale* and *B. bigemina*. This is the first report in America of *R. rickettsii* in American bison. American bison has been described as an important reservoir for pathogens of zoonotic and veterinary importance; thus, the presence of tick-borne pathogen DNA in the JBR American bison indicates the importance of continuous wildlife health surveys.

## 1. Introduction

The American bison (*Bison bison*) is listed as near-threatened under the red list and is in danger of extinction in Mexico (“NOM-059-SEMARNAT-2010” 2010) [1]. Conservation efforts have allowed restoration of numbers, with four wild herds in North America [2], one present for a little less than a century in northern Mexico [3] at Janos, Chihuahua, and recently at Maderas del Carmen, Coahuila. However, the transmission of infectious diseases between wildlife and livestock is threatening wildlife conservation efforts [4]; as a result, some wildlife remains isolated [5] to avoid wildlife–domestic animal interactions. In North America, only 5% of the bison population remains in wild herds, while the rest are subject to animal production standards and are in close contact with humans and domestic cattle. Different zoonotic diseases have been reported in bison [6], and, given their close interactions with both humans and domestic animals, there are particular animal and human health concerns. Recent studies have demonstrated the presence of different emerging pathogens at the Janos Biosphere Reserve (JBR) [7,8]. Here, we report the molecular detection of tick-borne pathogens found in a wild herd of American bison located in the JBR. We used pathogen-specific polymerase chain reaction (PCR) assays, followed by DNA sequencing of purified amplicons, and finally performed a genetic distance analysis of Rickettsia and Babesia DNA found in bison blood.

The potential role of wildlife as a source of infection for domestic animals has been widely discussed [9]. Brucellosis, a non-vector-transmitted disease capable of causing abortions [10], has been detected in the Yellowstone bison population since 1917 [11]; many animals of this herd migrate to winter ranges, increasing the risk of wildlife–domestic contagions. Tick-borne diseases (TBDs) can be traced back well over 100 years [12]; currently, the incidence of TBDs is on the rise globally [13]. In Mexico, there are reports of TBDs in wild animals [14,15,16], including the state of Chihuahua [7,8]. Different TBDs in the Chihuahua state, specifically in JBR, have been reported, for instance, *Borrelia burgdorferi s. s.* was identified in *Ixodes* *kingi* [7] and *Rickettsia parkeri* in *Dermacentor* *parumapertus* ticks in jackrabbits [8]. In addition, *Ixodes soricis* [17], vector of Lyme borreliosis [18]; *Riphicephalus sanguineus* positive for *Theileria equi, Babesia caballi*, and *Anaplasma phagocytophilum* [19] and *Dermacentor albipictus* [20] vectors of Rickettsia have been reported in other regions of the Chihuahua state.

Among arthropod vectors, ticks transmit the greatest diversity of pathogens to humans, livestock, and companion animals [21]. The presence of TBDs depends on the geographical area, and the host availability [22]. There are two species belonging to the genus bison and there are TBD reports for both species; several tick-borne pathogens have been described in European bison (*Bison bonasus bonasus*) [23,24] and in American bison (*Bison bison*) in Canada and the United States [25,26] as well. *Dermacentor reticulatus* ticks collected from European bison carry *Rickettsia raoultii* and *Anaplasma phagocytophilum* [23,27]. Some of these agents have been eradicated in Poland thanks to extensive wildlife programs [28], but caution remains, as European bison is still considered a reservoir of different agents. *Anaplasma marginale* was reported in American bison in the United States and Canada [25,26], suggesting that Bison are potential reservoirs for TBDs. Furthermore, there is molecular evidence for *Borrelia burgdorferi sensu stricto* and *Rickettsia massiliae* in ticks collected from wild carnivores in the JBR [7] and *Rickettsia parkeri* in ticks on black-tailed jackrabbits (*Lepus californicus*). All this evidence calls for continuous monitoring of wildlife in the JBR, especially the American bison.

Previous studies in JBR suggest a multiple-host scenario regarding TBD. An accurate understanding of TBDs is essential to assess potential disease risks and the potential role of bison as a source of infection for cattle and humans. Our aim in this study was to document the presence of TBDs that affect cattle and humans in American bison inhabiting El Uno JBR in Chihuahua, Mexico.

## 2. Results

We collected bison blood from 26 American bison in the Janos Biosphere Reserve (JBR), and used pathogen-specific PCR assays of *Borrelia burgdorferi* s. l., *Rickettsia rickettsii*, *Babesia bovis*, *Babesia bigemina*, and *Anaplasma marginale,* followed by DNA sequencing of purified amplicons, and finally performed a genetic distance analysis of Rickettsia and Babesia DNA found in bison blood.

During animal inspection, no external parasites were observed. Bison’s extensive grooming behavior during fall [29] might reduce tick-infestation, however many authors suggest that bison are reservoirs of important pathogens [23]. The inspection for ticks was carried out while bison were in the chut for annual animal management; this in itself was stressful for the bison [30,31]. Therefore, inspection was very fast, and on several occasions it could not be complete, however, in the animals in which it was possible to check the ears, tail, torso, armpits and groin, no ticks were found. For all blood samples it was possible to obtain DNA that served as a template to amplify the G3PDH Glyceraldehyde-3-Phosphate Dehydrogenase (Housekeeping) (G3PDH) gene of the host bison indicating the absence of PCR inhibitors in DNA samples (Table 1) [32,33].

Nine of the 26 samples (34.6%) were positive for at least one pathogen; two samples (7.6%) yielded a co-infection for two different pathogens. Three animals (11.5%) yielded positive for *B. burgdorferi s. l.* (animal IDs 3, 16 and 18) detected by amplification of the Ly-1 gene form *Borrelia burgdorferi*, another three animals (11.5%) were positive for *R. rickettsii* (animal IDs 31, 40 and 45 all females) identified by amplification of ompA gene, and five more animals (19.2%) were positive for *B. bovis* (animal IDs 2, 3, 18, 22, and 32) identified by amplification of rap1 gene. There were no positives for *A. marginale* (msp5 gene) or *B. bigemina* (Spel-Aval gene). These results are shown in Table 1. Of the 26 animal samples, two individuals were co-infected with *B. burgdorferi s. l.* and *B. bovis*, representing a 7.69% co-infection rate (animal IDs 3 and 18).

**Figure 1:**
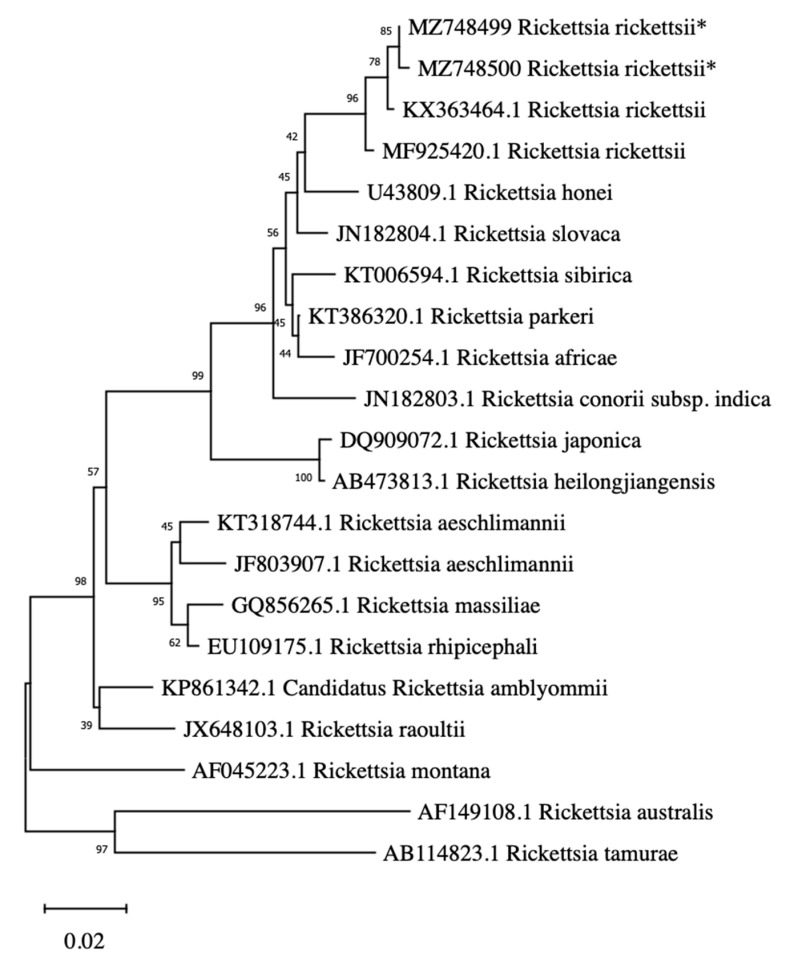
Genetic distance analysis of *Rickettsia rickettsii* identified in American bison (*Bison bison*) at Janos Biosphere Reserve, Chihuahua, Mexico (GenBank submission id: MZ748499 and MZ748500). Neighbor-joining phylogenetic tree (using K2P algorithm) was inferred using ompA gen of reference sequences of *Rickettsia* spp. The reliability of internal branches was assessed using the bootstrap test (1000 replicates). Scale bar indicates the proportion of nucleotide divergence. Asterisk (*) correspond to American bison samples.

**Figure 2:**
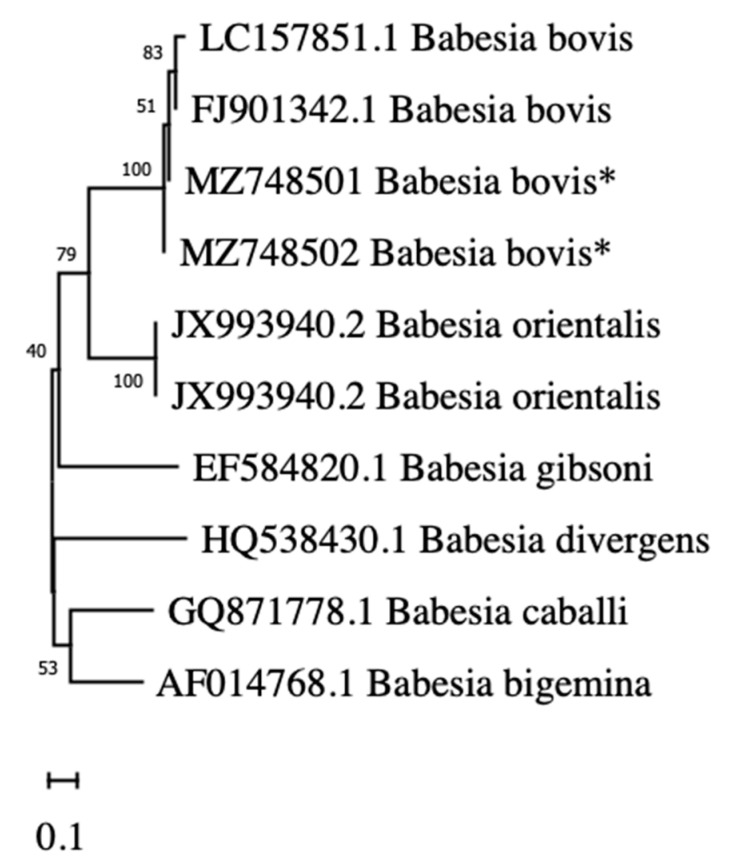
Genetic distances analysis of *Babesia bovis* identified in American bison (*Bison bison*) at Janos Biosphere Reserve, Chihuahua, Mexico (GenBank submission id: MZ748501 and MZ748502). Neighbor-joining phylogenetic tree (using K2P algorithm) was inferred using *rap-1* gene as reference sequences of *Babesia* spp. The reliability of internal branches was assessed using the bootstrap test (1000 replicates). Scale bar indicates the proportion of nucleotide divergence. Asterisk (*) correspond to American bison samples.

The ompA sequence obtained from ricketsias in American bison from JBR (GenBank submission id: MZ748499 and MZ748500) was 99% identical to 24 sequences of *R. rickettsii* obtained from GenBank. Additionally, the rap1 sequence obtained from babesias in American Bison (GenBank submission id: MZ748501 and MZ748502) was 99% identical to 70 sequences of *B. bovis* obtained in GenBank. The calculated genetic distances between ricketsias from bison from JBR were below 1% (0.2% and 0.4% respectively) when compared to *R. rickettsii,* and above 3% (3.1% and 3.3%) when compared to *R. parkeri*. Phylogenetic analysis confirmed that the genetic relationships of the sequences generated from American Bison and the reference sequences from GenBank formed well-defined groups with *R. rickettsi* and *B. bovis* (Figure 1 and Figure 2, respectively).

## 3. Discussion

Wild vertebrates are associated with several enzootic cycles of tick-borne pathogens contributing to the increase of ticks and TBDs in North America, playing an important role in the maintenance and transmission of zoonoses to livestock, humans, and other wildlife [34]. A more than two-fold increase in TBDs has been observed from 2004 (>22,000 cases) to 2016 (>48,000 cases) in the USA alone [35], and up to 476,000 people per year are infected with Lyme disease [36], with reports in both Mexico and USA [37,38,39,40] involving wild animals [41,42,43,44,45]. Furthermore, ticks and TBDs have evolved, adapting themselves to new vectors and vectors have adapted to new hosts; for instance, *Amblyoma immitator* has recently been proposed as a RMSF vector [46].

The main vectors for *B. burgdorferi* are ticks of the genus Ixodes, especially *I. scapularis*, *I. pacificus* and *I. ricinus*. A recent report in Mexico of ticks include *I. affinis*, which might be a competent vector for *B. burgdorferi* [47]. The main vectors for *R. ricketssi* are *Dermacentor variabilis*, *Ammblyoma americanum*, and *R. sanguineus* [48,49,50]. According to Levin et al., [48], *A. americanum* is one of the most aggressive ticks for humans, but this tick has not been accounted for in JBR, whereas *R. sanguineus* has been reported as a human-biting tick [51] and it has been reported in JBR [7]. Of 38 ticks collected from humans in Mexico [14], 6.3% of *A. cajennense* were positive for *A. phagocytophilum* and 10% of *D. variabilis* were positive for Rikcettsia. Current research on human TBDs may be unclear and disperse; however, from Borrelia (*B. afzelii*, *B. garrinii* y *B. burgdorferi s.s*.) infection reports between 1939–2020, the majority were in urban environments. Mexican states with the greatest number of reports of humans are northeast of Mexico, mainly Nuevo Leon and Tamaulipas [38]. Recently, an isolated case of *B. burgdorferi s. s*. was reported in Sinaloa [52]. Even when reports from Chihuahua state on Borrelia are limited, it is imperative to monitor human reports and wildlife, especially in rural areas where evidence might lack diffusion.

In the USA, Lyme disease is the most prevalent TBD [35,37]; the main vector in Mexico and USA are ticks of the genus *Ixodes* [9,53]. In this study, although inspection of the bison was carried out, we did not find any ticks. This finding is not conclusive; bison grooming behavior [29] and high levels of stress during handling routines [23] result in limited inspection, thus our findings do not exclude ticks using bison as hosts. Furthermore, in Mexico, in the south of Chihuahua state, ectoparasites in wild animals [17] have been identified as *Rhipicephalus sanguineus s. l*., *Dermacentor parumapertus*, *D. albipictus*, *Ornithodoros sp*. and *Ixodes sp*. in wild carnivores [7,20,39]. While Ixodes is an important pathogen vector for Lyme there is still research on Ornithodoros, and Dermacentor appears to be relevant for elk only [54].

The highest prevalence registered in this study corresponded to *B. burgdorferi s. l*., with 19.2% (n = 4). This disease has previously been reported in JBR in other species, such as wild carnivores and domestic animals [7]. Colunga-Salas et al. [38], reviewed cases reported in Mexico from 1930 to 2020; 29.5% were reports on humans, while 70.5% reported an animal species. They identified six *Borrelia* species: three responsible for Relapsing Fever (*B. duguessi*, *B. mazzottii* and *B. turicatae*) and three more are responsible for Lyme borreliosis (*B. afzelii*, *B. burgdorferi s. s*. and *B. garini*). These were identified with specific diagnostic tests (ELISA, IFA, PCR, microscopy and clinical laboratory diagnostics) in domestic (dog, horse, bull), wild species (deer, cougar, white-throated woodrat, field mouse, fox, rabbit among others) and humans, in 18 out of 32 states, including Chihuahua. *Borrelia burgdorferi s. l.* was reported in European bison (*B. bonasus*) with a prevalence of 13.33% [45], and 3.3% [55] similar to that found in the present work. While *B. burgdorferi* is relatively transient in blood and its vector *I. scapularis* does not parasite cattle, climate change has modified tick behavior and it has been suggested that ticks can parasite non-usual hosts [56]. Furthermore, the fact that *B. burgdorferi s.l.* DNA was detected in the blood from 13.33% of wild bison suggests that these animals are an important host of this spirochete [45]. Primers used in this study proved to be useful for the diagnosis of Lyme disease [57], although they could amplify other spirochetes [58].

Another zoonotic etiological agent suspected in the present work was *Rickettsia rickettsii*, the causal agent responsible for RMSF, and this is the first report worldwide of this agent in bison. There is evidence of several *Rickettsia* species found in different wildlife species [45,59]. *Rickettsia ricketsii* vectored by the tick *Rhipicephalus sanguineus* is the most relevant for public health. Wildlife particularly might be potential reservoirs, given that monitoring of such species is often sporadic and there is little veterinary management, yet humans commonly have indirect contact with wildlife by sharing environments, either accidentally or intentionally.

In bison blood samples, sequences with 99% identity to *R. rickettsii* were obtained. We only obtained the partial sequence of the OmpA gene and even while it might be necessary in the future to amplify other genes, genetic distance between the two obtained rickettsial DNA in this study compared to *R. rickettsii* is below 1%. Phylogenetic studies comparing isolated *R. parkeri* suggest that interspecific percent variation is under 2% [60]. In fact, reference sequences from GeneBank show a genetic distance below 1% with *R. rickettsii*, whereas genetic distance from our samples with *R. parkeri* is above 3%, suggesting that DNA samples in this study might belong to *R. rickettsii* [61,62]. Similarly, Ortega-Morales [63] compared *R. rickettsii* with other rickettsias and reports based on the OmpA gene. Further studies detecting different genes such as ribosomal RNA and citrate synthase genes might be necessary to corroborate this finding.

The finding of *Babesia bovis* DNA in American bison blood is also particularly important. Tick fever or cattle fever (babesiosis) is caused by parasites of the genus *Babesia*; the most important pathogens for cattle are *B. bovis* and *B. bigemina*, DNA of the latter was not identified in this work. Losses caused by babesiosis include mortality, abortions, slow weight gain or low milk production, and costs associated with control measures such as vaccines, treatments and ectoparasite control [64]. Thanks to the work of the US Cattle Fever Tick Eradication Program (CFTEP) in 1943, *R. microplus* and *R. annulatus*, vectors of these diseases, were eradicated in the United States, giving the status of babesiosis-free to cattle in that country [65]. However, in Mexico up to 75% of cattle are at risk of suffering from the disease [66,67]. In addition, it is estimated that these ticks are present in more than half of the national territory [68]. However, Chihuahua is considered a common-cattle tick-free state, except for the south municipalities of Morelos and Guadalupe y Calvo. Therefore, neither *Rhipicephalus microplus* nor *Rhipicephalus annulatus* would be expected to occur in the study area. To the best of the authors’ knowledge, there is no current report in the literature which clearly substantiates that the common cattle tick can infest the American bison. Nonetheless, a report of the Committee on Parasitic Diseases of the United States Animal Health Organization indicated that the “continued ingress of fever ticks from northeastern Mexico on cattle, equines, White-tailed deer, nilgai, America elk, bison, and axis deer” to the USA was one of the major eradication program issues of the CFTEP in 2007 [69].

On the other hand, while Babesia parasites can also be transmitted mechanically between animals when small amounts of blood are transferred on reused, non-sterilized needles or field surgical instruments or by biting flies [70], and there is evidence that American bison can be clinically affected by *Babesia bovis*, [71], *B. bigemina* [72], and *B. major* [73], animals detected PCR positive in this study remained clinically healthy. Unfortunately, there are no reports currently available on the prevalence rates of Babesia infection in cattle or other wild ungulates such as deer in the area of study which are much needed, and efforts should be dedicated to overcome this lack of important epidemiological data. *Babesia divergens* (the European cattle Babesia) was recently detected in three out of 37 (8%) tissue samples (spleen and muscle) of European bison (*Bison bonasus*) in Lithuania [74]. Therefore, and similarly to what was described for *R. rickettsii*, further analysis of other genes will be required to ascertain the presence of this pathogen of veterinary importance. Here we identified *B. bovis* (19.2%), implying that the pathogen might be present in the area; thus, bison that coexist with animals and domestic livestock, as well as other wild animals, could facilitate the circulation of pathogens. Furthermore, babesiosis has also been reported in Mexico using different analysis, in both water buffalo (*Bubalus bubalis*) [75] and exotic deer: fallow deer (*Dama dama*) and axis deer (*Axis axis*) [15]. Prevalence rates observed in these studies varied depending on the diagnostic method used, suggesting a sensitivity/specificity issue. Presence of babesiosis in wildlife in Northern Mexico takes on particular importance because breeding and production of beef cattle are carried out mainly on extensive farms where cattle are in close proximity to wildlife, facilitating the exchange of ticks between wild species and livestock [76,77].

Other countries in America have identified babesiosis in domestic cattle, wildlife, and water buffalo, and diagnosis with PCR and ELISA vary from 0.6% up to 50% [78,79]. Furthermore, Northern Botswana (Africa) reports the presence of hemoparasites transmitted by ticks in African buffalo (*Syncerus caffer*), the highest prevalence observed was for *Theileria parva* (60%) and *Theileria mutans* (37%), while for the other pathogens, prevalence of 30% for *A. marginale* subsp. centrale, 20% for *A. marginale*, 23% for *Babesia occultans* and finally 6% for *Ehrlichia ruminantium* were found [80]. Although results are different from those found here, all these are pathogens that naturally affect livestock species in that country, suggesting that wildlife and domestic animals participate in the process of vector transmission of pathogens, as proposed by other authors [81,82,83].

Bovine anaplasmosis, whose etiological agent is *A. marginale*, is an endemic disease of tropical and subtropical regions of worldwide distribution that also causes significant economic losses in livestock, and together with *Anaplasma phagocytophilum* are the two rickettsiae of the Anaplasmataceae family of greatest importance to public and veterinary health [84]. Bovine anaplasmosis has already been described in American bison from USA and Canada [25,83], water buffalo in Cuba [78], and *A. phagocytophilum* in European bison (*B. bonasus*) [27]. The presence of *A. marginale* has been reported in cattle in different regions of Mexico [84,85,86] and although the causal agent was searched for in the present study, it was not possible to identify it. However, this finding does not imply that the disease is not present, it is possible that animals did not display ricketsemia during sample collection, hence the importance of continuous monitoring.

It is thus important to continue and extend studies to detect vector-borne diseases, both in domestic and wild animals that also function as sentinels for zoonotic diseases. The interaction between the different animal species and humans can favor the distribution of both emerging and re-emerging diseases, making their detection and management more complex [87]. Our results of tick-borne pathogen DNA in bison in the JBR suggest an increased risk of tick-borne diseases at the domestic-wildlife interface.

## 4. Materials and Methods

**Figure 3:**
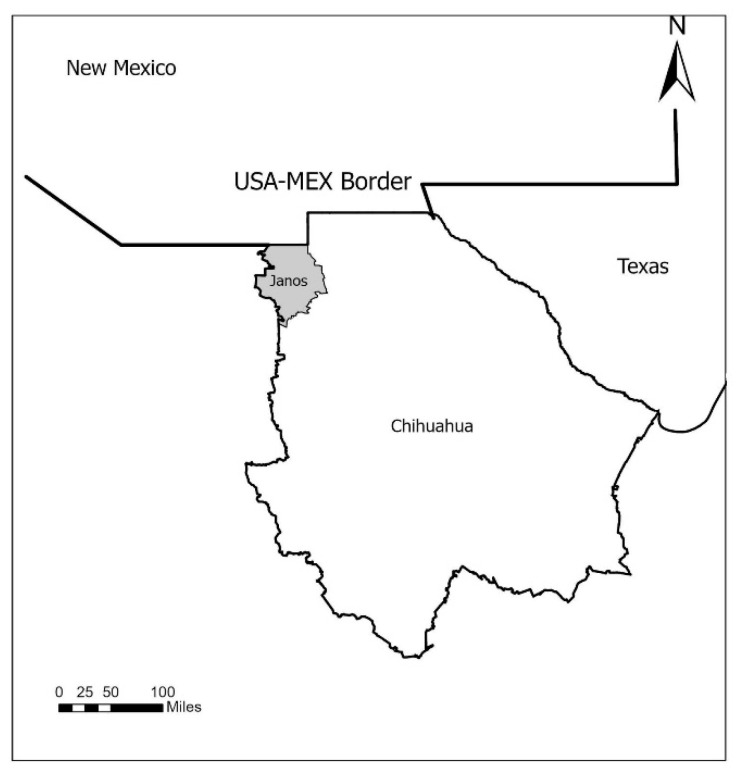
Localization of Janos Biosphere Reserve found at the US-Mexican border in the Chihuahuan state south of New Mexico.

**Figure 4:**
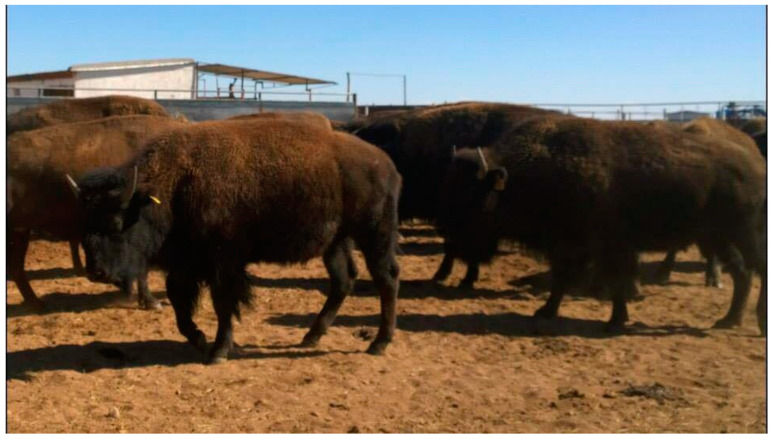
American bison (*Bison bison*) at Janos Biosphere Reserve.

**Figure 5:**
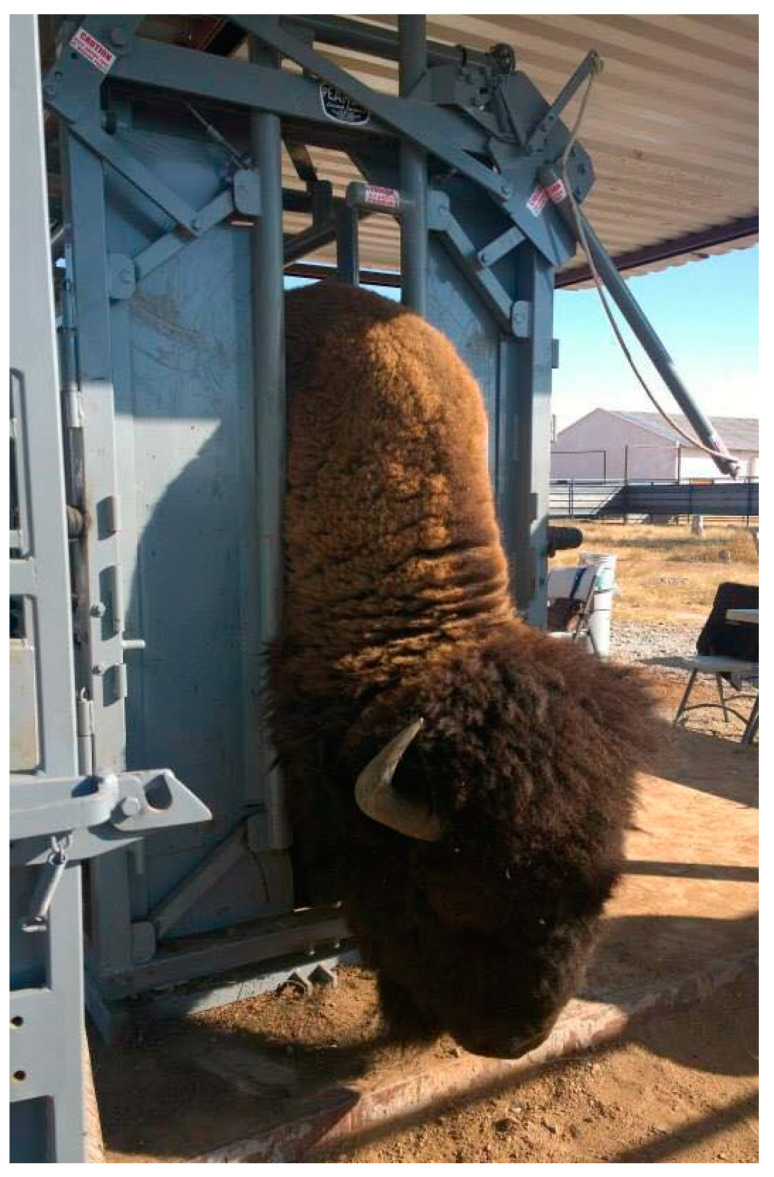
American bison (*Bison bison*) during the handling routine.

American bison sampling was performed during October 2014 at the JBR in Chihuahua located at 31°11′7.6344″ N, 30°11′24.4548″ N latitude and 108°56′49.1712″ W, 108°56′22.0992″ W, longitude. JBR is located south of New Mexico, around 70 miles south of the USA-Mex border (Figure 3), and it is composed primarily of native grasslands that transform into wooded mountain range at higher elevations. During collection, the reserve housed 66 bison counting adults and juveniles (Figure 4). Blood collection was conducted during routine veterinary management. Animals were restrained using a squeeze chute, and following a medical examination, a physical exploration for ectoparasites was performed (Figure 5). Given the high stress levels in the chute and the challenging handling of big individuals, we collected samples only from 26 individuals at random, about 36.36% of the present population. Furthermore, the physical exploration included both visual examination and a physical search behind the ears, neck, under the tail, torso, and commonly tick-infested areas.

Bison blood samples were then collected by jugular puncture of the animals using vacutainer tubes with EDTA, which were stored at 4 °C until processing in the laboratory. Once in the laboratory, the cell pack was separated from whole blood by centrifugation (10 min × 3000 rpm), then samples were frozen at −80 °C until molecular analysis to detect the presence of pathogens transmitted by ticks. Before DNA isolation, 200 µL of each sample was taken and washed three times with 0.15% saponin solution [88].

Laboratory analysis was conducted at the Laboratory of Veterinary Clinical Pathology and Molecular Biology of the Autonomous University of Ciudad Juárez (LPCV-UACJ) and the National Disciplinary Research Center on Animal Health and Safety (CENID-SAI, INIFAP). The animal handling protocol was revised and approved by the Secretary of Environment and Natural Resources in Mexico SGPA/DGVS/01610/16 and handling complied with Mexican and American guidelines for animal research (Guide for the Care and Use of Laboratory Animals in National Resource Council, 2011).

### 4.1. Genomic DNA Extraction and Nested PCR

Genomic DNA extraction was performed with a commercial kit (Ultra Clean DNA Blood Spin^®^, MoBio, Carlsbad, CA, USA) following the manufacturer’s directions. Every genomic DNA extraction from bison blood samples, as well as PCR protocols for pathogens of interest, were duplicated in two different laboratories, CENID and UACJ.

To confirm DNA presence, a first PCR with Glyceraldehyde-3-Phosphate Dehydrogenesa (G3PDH) was conducted. Once amplifiable DNA was confirmed; bison DNA samples were analyzed by nested PCR for molecular detection of *R. rickettsii*, *B. bovis*, *B. bigemina*, *Anaplasma marginale*, and by single PCR for *B. burgdorferi s. l.* (Table 2). Each reaction was prepared to a final volume of 25 µL and amplified in an endpoint thermal cycler (BIO-RAD^®^ C-1000 Touch, Hercules, CA, USA) with the protocols previously reported (Table 2) [89,90,91,92,93]. The first PCR amplification reactions contained 12.5 µL Go Taq Green Master Mix, 2× (400 µM of dNTP, 3 µM MgCl_2_ and 1.5 U *Taq* DNA polymerase in buffer 2× pH 8.5, PROMEGA^®^, Madison, WI, USA), 1 µL of each outer primer (10 pmol), 5 µL extracted DNA sample and 5.5 µL PCR-grade water. The nested PCR was performed under the same conditions as above, except that 2 µL of the amplicon generated in the first amplification and 8.5 µL PCR-grade water were used. Amplicons were analyzed by 2% agarose gel electrophoresis containing ethidium bromide and visualized by UV transillumination (BioDoc-It, UVP LLC^®^, Upland, CA, USA).

Positive controls of *B. burgdorferi s. l.* and *R. rickettsii* were donated by Dr. Luis Tino-co-Gracia and all were previously sequenced. Positive controls of *B. bovis*, *B. bigemina,* and *A. marginale* were obtained from CENID-SAI, INIFAP. For all positive controls, we used genomic DNA extracted from individually infected samples. As negative controls during PCR reactions, we used nuclease-free water along with uninfected bovine genomic DNA since uninfected bison DNA was not available.

### 4.2. DNA Sequencing

Amplicons were purified from the agarose gel using a commercial kit (Wizard^®^ SV Gel and PCR Clean-Up System, PROMEGA^®^, Madison, WI, USA) and sent out for sequencing at Biotechnology Institute of National Autonomous University of Mexico. Nucleotide sequences were then compared with the NCBI database employing the BLAST program available online (http://www.ncbi.nlm.nih.gov/BLAST/ accessed on 14 August 2021).

### 4.3. Phylogenetic Analysis

For distance genetic analysis between species, neighbor-joining (NJ) phylogenetic trees based on sequences of *Rickettsia* spp. ompA gene and *Babesia* spp. RAP-1 gene were inferred using Molecular Evolutionary Genetics Analysis (MEGA) software, X v version [93]. Reference sequences of *Rickettsia* (using ompA) and *Babesia* (using RAP-1) species or strains, from diverse hosts were collected in GenBank to build the NJ tree [63]. The sequences were aligned using MUSCLE algorithms configured applying the cluster method of NJ with 1000 interactions. For reconstruction of NJ trees, the Kimura 2- parameter model [94,95] was used. Bootstrap values to test the robustness of the tree were obtained by conducting 1000 replications [94].

## 5. Conclusions

The DNA findings of the present work show that wild animals might be carriers and reservoirs of pathogens that affect domestic animals, and humans, including some zoonotic diseases, and suggest that they play an important role in their transmission. This is the first molecular detection of Rickettsia *rickettsii* DNA in American bison. However, it is still necessary to monitor livestock herds as well as different wild animals that cohabit the area. In addition, the DNA detection of pathogens transmitted by ticks suggests that ticks also parasitize bison; therefore, continuous monitoring is suggested, even looking for other TBDs such as those of viral origin. Furthermore, collaborative studies along the US-Mexico border are necessary given the reports of vectors and high prevalence relevant in the wild-domestic-human interface.

## Figures and Tables

**Table 1:** Results obtained in the PCR assays for the pathogens of interest.

Animal ID	*Gene* *G3PDH*	*Borrelia* *burgdorferi* *s. l.*	*Rickettsia* *rickettsii*	*Babesia* *bovis*	*Babesia* *bigemina*	*Anaplasma* *marginale*
2	+	−	−	+	−	−
3	+	+	−	+	−	−
4	+	−	−	−	−	−
5	+	−	−	−	−	−
8	+	−	−	−	−	−
9	+	−	−	−	−	−
10	+	−	−	−	−	−
12	+	−	−	−	−	−
14	+	−	−	−	−	−
16	+	+	−	−	−	−
18	+	+	−	+	−	−
20	+	−	−	−	−	−
22	+	−	−	+	−	−
24	+	−	−	−	−	−
25	+	−	−	−	−	−
27	+	−	−	−	−	−
30	+	−	−	−	−	−
31	+	−	+	−	−	−
32	+	−	−	+	−	−
34	+	−	−	−	−	−
38	+	−	−	−	−	−
40	+	−	+	−	−	−
44	+	−	−	−	−	−
45	+	−	+	−	−	−
47	+	−	−	−	−	−

**Table 2:** Sequences of primer sets, and protocols used for PCR detection.

Pathogen	Oligonucleotide Sequence (5′–3′)	Product Size (bp)	PCR Protocol	References
Glyceraldehyde-3-Phosphate Dehydrogenase (Housekeeping)	GAPDHF-CCTTCATTGACCTCAACTACAT GAPDHR-CCAAAGTTGTCATGGATGACC	400	94 °C for 5 min initial denaturation, followed by 35 cycles of 94 °C for 1 min, 55 °C for 1 min, 72 °C for 1 min, then 72 °C for 15 min for the final elongation	[32]
*Borrelia burgdorferi s. l.*	LY2F-GAAATGGCTAAAGTAAGCGGAATTGTAC LY2R-CAGAAATTCTGTAAACTAATCCCACC	231	94 °C for 4 min initial denaturation, followed by 40 cycles of 94 °C for 45 s, 55 °C for 45 s, 72 °C for 45 s, then 72 °C for 7 min for the final elongation	[89]
*Rickettsia* spp. (*ompA*)	Rr190.70P-ATGGCGAATATTTCTCCAAAA Rr190.701N-GTTCCGTTAATGGCAGCATCT	631	95 °C for 5 min initial denaturation, followed by 35 cycles of 95 °C for 30 s, 58 °C for 30 s, 65 °C for 45 s, then 72 °C for 7 min for the final elongation	[90]
*Rickettsia rickettsii* (*ompA*)	Rr190.70P-ATGGCGAATATTTCTCCAAAA Rr190.602N-AGTGCAGCATTCGCTCCCCCT	532	96 °C for 30 s initial denaturation, followed by 35 cycles of 94 °C for 30 s, 58 °C for 30 s, 72 °C for 45 s, then 72 °C for 7 min for the final elongation	[90]
*Babesia bovis* (*rap*-1)	BOF-CGAGGAAGGAACTACCGATG BOR-GGAGCTTCAACGTACGAGGT	354	95 °C for 5 min initial denaturation, followed by 35 cycles of 95 °C for 1 min, 55 °C for 1 min, 73 °C for 1:30 min, then 72 °C for 15 min for the final elongation	[91]
*Babesia bovis* (*rap*-1)	BOFN-TGGCTACCATGAACTACAAGACTTA BORN-GAGCAGAACCTTCTTCACCAT	275	95 °C for 5 min initial denaturation, followed by 35 cycles of 95 °C for 1 min, 55 °C for 30 s, 73 °C for 1:30 min, then 72 °C for 15 min for the final elongation	[91]
*Babesia bigemina* (*SpeI-AvaI*)	BIF-CATCTAATTTCTCTCCATACCCC BIR-CCTCGGCTTCAACTCTGATGCC	278	95 °C for 5 min initial denaturation, followed by 35 cycles of 95 °C for 1 min, 65 °C for 1 min, 73 °C for 1:30 min, then 72 °C for 15 min for the final elongation	[91]
*Babesia bigemina* (*SpeI-AvaI*)	BIFN-CGCAAGCCCAGCACGCCCCGGT BIRN-CCGACCTGGATAGGCTGTGATG	170	95 °C for 5 min initial denaturation, followed by 35 cycles of 95 °C for 1 min, 65 °C for 30 s, 73 °C for 1:30 min, then 72 °C for 15 min for the final elongation	[91]
*Anaplasma* spp. (*msp*5)	MSP5F-ACCTTCTGCTGTTCGTTGC MSP5R-TGTACCACTGCCATGCTTAAG	628	95 °C for 3 min initial denaturation, followed by 35 cycles of 95 °C for 30 s, 65 °C for 1 min, 72 °C for 1 min, then 72 °C for 10 min for the final elongation	[92]
*Anaplasma marginale* (*msp*5)	MSP5FN-CATAGCCTCCGCGTCTTT MSP5RN-CTTAAACAGCTCCTCGCCTT	466	95 °C for 3 min initial denaturation, followed by 35 cycles of 95 °C for 30 s, 65 °C for 1 min, 72 °C for 1 min, then 72 °C for 10 min for the final elongation	[92]

## Data Availability

Data are available from the authors upon reasonable request.
